# The effect of a motivational intervention on weight loss is moderated by level of baseline controlled motivation

**DOI:** 10.1186/1479-5868-7-4

**Published:** 2010-01-22

**Authors:** Kelly H Webber, Jeanne M Gabriele, Deborah F Tate, Mark B Dignan

**Affiliations:** 1 Department of Nutrition and Food Science, University of Kentucky, Lexington, KY, USA; 2 Department of Psychology and Mental Health, G.V. (Sonny) Montgomery VA Medical Center, Jackson, MS, USA; 3 Department of Nutrition and Department of Health Behavior and Health Education, The University of North Carolina at Chapel Hill, Chapel Hill, NC, USA; 4 Prevention Research Center, University of Kentucky, Lexington, KY, USA

## Abstract

**Background:**

Clinic-based behavioral weight loss programs are effective in producing significant weight loss. A one-size-fits-all approach is often taken with these programs. It may be beneficial to tailor programs based on participants' baseline characteristics. Type and level of motivation may be an important factor to consider. Previous research has found that, in general, higher levels of controlled motivation are detrimental to behavior change while higher levels of autonomous motivation improve the likelihood of behavior modification.

**Methods:**

This study assessed the outcomes of two internet behavioral weight loss interventions and assessed the effect of baseline motivation levels on program success. Eighty females (*M *(*SD*) age 48.7 (10.6) years; BMI 32.0 (3.7) kg/m^2^; 91% Caucasian) were randomized to one of two groups, a standard group or a motivation-enhanced group. Both received a 16-week internet behavioral weight loss program and attended an initial and a four-week group session. Weight and motivation were measured at baseline, four and 16 weeks. Hierarchical regression analysis was conducted to test for moderation.

**Results:**

There was significant weight loss at 16-weeks in both groups (*p *< 0.001); however there were no between group differences (*p *= 0.57) (standard group 3.4 (3.6) kg; motivation-enhanced group 3.9 (3.4) kg).

Further analysis was conducted to examine predictors of weight loss. Baseline controlled motivation level was negatively correlated with weight loss in the entire sample (r = -0.30; *p *= 0.01). Statistical analysis revealed an interaction between study group assignment and baseline level of controlled motivation. Weight loss was not predicted by baseline level of controlled motivation in the motivation-enhanced group, but was significantly predicted by controlled motivation in the standard group. Baseline autonomous motivation did not predict weight change in either group.

**Conclusions:**

This research found that, in participants with high levels of baseline controlled motivation for weight loss, an intervention designed to enhance motivation for weight loss produced significantly greater weight loss than a standard behavioral weight loss intervention.

## Introduction

Current estimates suggest that approximately 32% of United States (US) adults are obese, with a body mass index (BMI) of 30 or greater, and 34% of adults are overweight, with a BMI of 25.0 to 29.9 [1,2]. Preventing and treating obesity is a multifaceted problem that will likely need to be addressed on multiple levels ranging from policy to individual interventions. On the individual level, successful weight loss programs exist and the most beneficial are clinic-based face-to-face behavioral programs with weekly visits [3,4]. Although effective, these programs tend to be costly, inaccessible to some, and inconvenient due to the time required and the need to travel to a clinic. These limitations decrease the potential public health impact of these programs. As a result, Internet-based weight loss programs, which have the potential to reach larger numbers of individuals, potentially at a lower cost, have become more common.

Self-directed Internet programs are effective in producing an average weight loss of 5.5 kg in the first six months of treatment compared to 8-10 kg of weight loss seen in face-to-face programs [3-5]. More frequent program utilization has been associated with better weight loss outcomes in Internet-based programs; however, over time utilization decreases [6,7]. This decreasing utilization could be related to decreasing motivation levels. In an effort to improve outcomes in self-directed programs, participant motivation could be targeted and enhanced.

Self-Determination Theory (SDT) suggests that there are two different types of motivation, autonomous and controlled [8]. Autonomous motivation is a measure of a person's internal or personal reasons for change, including all intrinsic reasons for change and some extrinsic reasons. Controlled motivation is a measure of the extent to which a person feels external pressures to change, similar to extrinsic motivation [9]. SDT suggests that greater autonomous motivation is associated with greater likelihood of behavior change and high controlled motivation is associated with less likelihood of success with change [9]. Previous research indicates that autonomous motivation is positively associated with greater physical activity and fruit and vegetable consumption; whereas, controlled motivation has no association or a negative association with these outcomes [10,11]. Additionally, higher levels of autonomous motivation measured five to ten weeks into a weight loss program has been predictive of better 6-month weight loss and 23-month weight maintenance while greater controlled motivation was predictive of less weight loss at six months [6].

These motivational constructs may help us to gain an understanding of when and for whom our weight loss programs work. Because autonomous motivation is influential to physical activity, dietary behavior, and weight management, finding ways to increase autonomous motivation may be important for weight loss success and long term weight loss maintenance.

SDT suggests several ways in which motivation may be positively influenced. Autonomy, competence, and relatedness are the three central psychological needs specified by SDT. The support of these three needs can lead to greater autonomous regulation and motivation [9]. Deci and Ryan, the authors of SDT, suggest that motivational interviewing (MI) [12], a type of counseling style, is conducive to supporting these three basic needs. The use of MI in various disciplines has led to improved program attendance, adherence, and retention [13-16]. At least one study has found that the use of MI principles in a weight loss program may lead to increases in autonomous motivation over time [7]. Studies which have used MI techniques in weight loss treatments have shown mixed results on weight loss and none of these previous interventions have reported on motivation levels of participants at baseline or throughout the program [17-19].

Additionally, there are other intervention components that could be used to enhance autonomous motivation and improve weight loss. A sense of personal competence, or self-efficacy, can be enhanced through the accomplishment of small achievable goals [20,21]. Therefore, personal goal setting in an autonomy supportive climate might also improve autonomous motivation. Finally, Deci and Ryan also suggest that greater mindfulness is associated with greater autonomous regulation [9]. It is proposed that greater mindfulness may allow for integration of introjected or controlled reasons for change into a more autonomous mindset, thus leading to a decrease in controlled motivation and an increase in autonomous motivation. Therefore, an intervention component that encourages mindfulness might also increase autonomous motivation and weight loss.

The aim of the present study was to determine if a motivation-enhanced behavioral weight loss intervention, which incorporated principles of MI, personal goal setting, and journaling, resulted in greater weight loss, greater program usage, and greater increases in autonomous motivation than a standard behavioral weight loss program. An additional aim was to determine whether baseline levels of autonomous and controlled motivation moderated the effect of the two interventions on weight loss.

## Methods

### Participants

Adult women ages 22-65 who had a body mass index (BMI) between 25 and 40, and home access to a computer with Internet service were recruited. Exclusion criteria included a medical diagnosis of orthopedic or joint problems that might prohibit regular exercise, hospitalization for a psychiatric disorder within the last year, history of anorexia or bulimia nervosa, intention to move out of the immediate area within the study period, medical diagnosis of HIV, diagnosis with a major psychiatric disorder (i.e. bipolar disorder or schizophrenia), pregnant, nursing, or planning to become pregnant within the study period, less than nine months post-partum, cancer diagnosis within five years with the exception of skin cancer, and recent weight loss of ≥ 10 pounds. Exclusion criteria also included the endorsement of any of the first three items on the Physical Activity Readiness Questionnaire (PAR-Q) [22,23] which included heart problems, chest pain, faintness, or dizzy spells, or endorsement of any of the other items on the PAR-Q without a physician's consent. Twenty-two participants required physician's consent, which consisted of a signed form returned to study staff that stated that the individual's physician approved of the individual's participation in the study.

**Figure 1:**
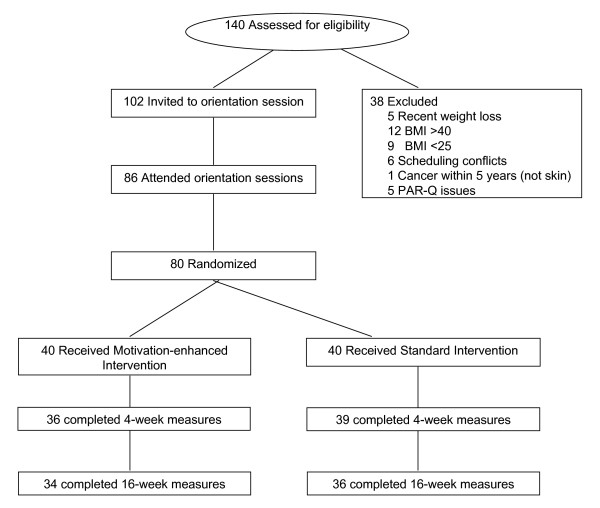
**Participant Flow Diagram**.

Participants were recruited through a newspaper advertisement and a university staff listserv and were screened for eligibility via telephone interview. Eligible participants were then invited to a study information session. At this session, participants learned further details about the study and interested participants were asked to sign a consent form. Participants then returned for a baseline assessment visit and completed baseline questionnaires (Figure 1).

### Study design

After baseline assessments were completed, participants were randomized to one of two study groups, Standard treatment (n = 40) or Motivational treatment (n = 40). Participants then attended a baseline group face-to-face session at the university and received access to the study website. Participants were asked to return for a face-to-face group session at four-weeks and a follow-up assessment visit at 16 weeks. All study procedures were approved the University of Kentucky Institutional Review Board (IRB).

### Intervention content

Both groups received a separate face-to-face two-hour weight loss session at the beginning of the study led by a dietitian trained in MI by the Motivational Interviewing Network of Trainers (MINT). The session took place in a classroom at the university. All participants were given equivalent instructions for diet and exercise during the face-to-face weight loss sessions. This information was based on the Diabetes Prevention Program and the recommendations of the Dietary Guidelines for Americans 2005 [24], the American College of Sports Medicine [25], and The Institute of Medicine (IOM) report on Dietary Reference Intakes [26]. The participants' were instructed to eat a low-calorie and low-fat diet of not more than 25% of calories from fat and not more than 1200 kcal/day for participants weighing less than 200 pounds and a diet of 1500 kcal/day for participants weighing 200 or more pounds. Thirty to sixty minutes of moderate to vigorous physical activity per day was also recommended for all participants.

The Standard group session included weight loss basics, diet and exercise recommendations, instructions on self-monitoring, and an overview of the study website but did not explicitly use motivational techniques. The Motivational group session was led in an MI style and included weight loss basics, diet and exercise recommendations, instructions on self-monitoring, an overview of the study website, a group discussion of the pros and cons of weight loss, the importance of weight loss, reasons for weight loss, and proper goal setting techniques. Motivational group participants were asked to choose a goal they would like to work towards for the next four weeks. The session ended with a journaling activity in which participants were asked to write for 10 minutes about a time in the future when they had accomplished their weight loss goals. Participants were then encouraged to continue journaling about the weight loss process throughout the study. All participants were provided with a calorie book and self-monitoring diaries.

At the conclusion of the baseline face-to-face session, participants were given access to the password protected study website. The website contained weekly weight loss tips, weekly lesson postings, weekly recipes, a message board feature, and links to self-help diet, exercise, and behavioral modification resources available on the web. The site also had a link to a personal on-line self-monitoring report form which participants were asked to use to report, at least weekly, daily caloric intake and daily exercise. The website was identical for the two groups with the exception of separate message boards.

Topics for the 16-week behavioral weight loss lessons were similar to the core of the Diabetes Prevention Program (DPP) [27]. The DPP lessons were developed for use in a face-to-face individual setting where counselors interacted with participants and provided problem solving and support verbally. These lessons were modified for use in this study to be more self-directed and suitable for posting to the website and have been used in a previous study [7].

The week four group sessions lasted for approximately 60 minutes and differed in content for the two study groups. The Standard group received information on the health benefits of whole grains, how to read a food label, and ideas for incorporating more whole grains into their diet. The Motivational group session was led in a MI style and included a review of proper goal setting techniques, a discussion of goal achievement, a goal setting activity, and a guided journaling activity in which participants were asked to write for 10 minutes about a weight loss related personal accomplishment.

### Measures

Body weight was measured at baseline, four and 16 weeks by trained research assistants, blinded to group assignment. Participants' body composition was also assessed at baseline and sixteen weeks with the Life Measurement, Inc. Bod Pod, model 2007A (Concord, CA). Weight measurements at baseline and 16 weeks were taken with the Bod Pod scale. Weight at week four was measured by a calibrated Tanita medical digital scale, model # BWB-800A (Arlington Heights, IL). Participant's height was measured at baseline only. Participants wore a bathing suit or bike shorts and sports bra with no shoes for weight and height measurements at baseline and 16 weeks and street clothes for weight measurements at four weeks. Waist circumference was measured at the umbilicus.

In order to assess the impact of the intervention on dietary intake, the 2005 version of the Block Food Frequency Questionnaire (FFQ) [28] was administered at baseline and 16-week follow-up. A self-report measure of physical activity, based on questions used in the Women's Health Initiative (WHI), was administered at baseline and 16-week follow-up [29].

The following psychosocial variables were assessed at baseline and examined to determine possible between group differences. Weight loss self-efficacy was measured using the weight efficacy lifestyle questionnaire (WEL-Q) [30]. Exercise self-efficacy was measured using the Marcus 5-item exercise self-efficacy scale [31]. Depression was measured using the CES-D [32]. Social support was measured using the Multidimensional Scale of Perceived Social Support [33]. Weight loss motivation was measured using the autonomous and controlled regulation subscales of the Treatment Self-Regulation Questionnaire (TSRQ) for weight loss treatment [6,34,35] at baseline and again at four and sixteen weeks. All surveys were self-administered.

Self-monitoring was measured by online food and exercise diary forms. Completion of at least five days of monitoring was required for a participant to receive credit for monitoring in a given week. The number of logins to the website and the number of postings to the message boards were monitored and recorded.

### Statistical analysis

This study was designed to have at least 80% power to detect a difference of 2.5 kg between groups with a pooled standard deviation of 4.5 kg and alpha = 0.05 on the primary outcome of weight loss. All analyses were performed using Windows version 16.0 of the Statistical Package for the Social Sciences (SPSS, Chicago, IL). Descriptive statistics were used to characterize both study groups at baseline. Categorical variables were analyzed using Pearson chi-square analysis.

Univariate analysis of variance with a 0.05 significance level was used to assess whether treatment condition had an effect on pre-post changes in weight, waist circumference, and body fat percentage. Changes in outcome variables over time were examined using paired t-tests. Baseline predictors of weight loss were first examined using the Pearson R coefficient. Hierarchical regression analyses were conducted to test for moderation [36].

## Results

### Participants

Of the final participants, 62 were recruited through the newspaper advertisement and 18 were recruited through the employee listserv. Numbers from each recruitment source were evenly distributed across study groups (31 from newspaper and 9 from listserv in each group). All participants were female and 91% were Caucasian. Thirty-five percent of participants had a graduate degree or equivalent. The average age of participants was 48.7 (10.6) years and baseline BMI averaged 32.0 (3.7) kg/m^2^. At baseline, the two study groups did not differ on weight, percent fat mass, waist circumference, age, racial distribution, education, or number of children in the household, nor did they differ on the psychosocial measures of controlled or autonomous motivation, level of depression, self-efficacy for diet, self-efficacy for exercise, or social support. Caloric intake and expenditure did not differ between the groups at baseline either (Table 1).

**Table 1:** Baseline Measures.

	Standard Mean (SD)	Motivation-enhanced Mean (SD)	*p-value*
**Age**	47.9 (10.8)	49.5 (10.4)	0.51

**Race**	90% Caucasian	93% Caucasian	0.43

**Education**	68% College Graduate or Greater	85% College Graduate or Greater	0.06

**Weight (kg)**	84.2 (12.1)	84.3 (12.3)	0.97

**Waist Circumference (cm)**	96.5 (9.5)	97.3 (10.6)	0.73

**BMI (kg/m2)**	32.1 (3.6)	31.9 (3.9)	0.86

**Percentage** **Body Fat**	43.9 (5.1) %	44.6 (4.5) %	0.53

**Weight Loss Self-efficacy (WEL-Q)**	115.1 (26.2)	117.2 (26.8)	0.72

**Physical Activity Self-efficacy**	13.7 (3.4)	12.7 (3.0)	0.17

**Autonomous Motivation (TSRQ)**	6.1 (0.6)	6.1 (0.6)	0.76

**Controlled Motivation (TSRQ)**	2.6 (1.0)	2.4 (1.0)	0.46

**Social Support**	70.6 (16.0)	67.8 (15.3)	0.42

**Depression (CES-D)**	7.4 (5.6)	9.0 (8.2)	0.31

**Physical Activity** **(kcal/week)**	1008 (1026)	972 (894)	0.87

**Energy Intake** **(kcal/day)**	1872 (821)	1856 (598)	0.92

N = 80; participants did not differ on any baseline measure.

### Completers vs. Non-completers

Ninety-one percent of participants returned for the four-week weight measurements and 88% returned for 16-week follow-up (Figure 1). Loss to follow-up was due to the following: one person reported job loss and inability to travel to the center, one person reported difficulty with internet service, two people reported major family health problems, and six dropped out for unknown reasons. Those who completed the 16-week follow-up and those who did not return for 16-week follow-up did not differ on any baseline measure or study group assignment. There was no significant difference between weight outcomes when analyzed using completers only, last observation carried forward, or baseline carried forward, therefore the following analyses are for participants who completed the study (N = 70) (Table 2).

**Table 2:** Weight Change in Kg (SD) from Baseline to 16 Week

	Standard Group	Motivation-Enhanced Group	*p*-value for difference
Completers only (n = 70)	3.4 (3.6)	3.9 (3.4)	0.57

Intent-to-treat analysis (n = 80)	3.1 (3.5)	3.3 (3.4)	0.79

### Weight, waist circumference, and body fat change

Both groups lost weight from baseline to 16 weeks (*M *= 3.6 (3.5) kg; *p *< 0.001); however the there were no differences between the two study groups (*p *= 0.57). The Standard group lost 3.4 (3.6) kg and the Motivation-enhanced group lost 3.9 (3.4) kg (Table 2). Thirty-three percent of Standard group participants and 41% of Motivational group participants lost at least 5% of their initial body weight (*p *= 0.50). Waist circumference decreased from baseline to 16 weeks in both groups an average of 3.6 (5.2) cm and did not differ between the two groups (*p *= 0.99). Body fat percentage decreased between baseline and sixteen weeks in both groups an average of 1.7 (3.1) %, but did not differ between the two groups (*p *= 0.85) (Additional File 1).

### Lifestyle changes

There were no differences between groups in change in caloric intake, percentage fat intake, or physical activity (Additional File 1).

### Process Measures

Program usage was assessed through website visits, number of self-monitoring diaries completed, and number of posts to the message board. The Standard group visited the study website an average of 17.7 times while the Motivation-enhanced group visited the website 22.2 times (*p *= 0.32) over the 16-week study. The number of visits to the website was associated with weight loss in both groups (Standard r = 0.35, p = 0.04; Motivation r = 0.57, *p *< 0.001).

The average number of weekly diaries completed by the Standard group was 7.0 and the Motivation-enhanced group completed an average of 8.7 diaries (*p *= 0.20). The number of diaries completed was associated with weight loss in both groups (Standard r = 0.45, *p *= 0.01; Motivation r = 0.49, *p *< 0.01).

There were no differences between groups in the number of posts to the message board, 1.0 for the Standard group and 1.3 for the Motivation-enhanced group (*p *= 0.66). The number of posts to the website message boards was associated with weight loss in the Motivation-enhanced group only (r = 0.47, *p *= 0.01).

### Motivation Change

Between baseline and 16-week follow-up the Standard group significantly decreased in autonomous motivation from a value of 6.11 to 5.55 (*p *= 0.02) on a 7-point scale and the Motivation-enhanced group went from a value of 6.10 for autonomous motivation at baseline to a value of 5.63 (*p *= 0.10) at follow-up. There was no significant difference in the change score between the groups for autonomous motivation (*p *= 0.76).

### Additional Analysis

Levels of controlled and autonomous motivation at baseline and four weeks were assessed for correlations with 16-week weight change. Weight loss was negatively associated with baseline controlled motivation in the overall sample (r = -0.30; *p *= 0.01). At baseline, subjects with high controlled motivation were less likely to lose weight than their counterparts with low controlled motivation over the 16-week study. Additional analysis revealed that for the Standard group, controlled motivation at baseline was negatively related to weight loss (*r *= -0.54; *p *= 0.001). For the Motivation-enhanced group there was no relationship between controlled motivation and weight loss (*r *= 0.001; *p *= 0. 99). Baseline autonomous motivation was not correlated with weight change in the overall sample (r = 0.06; *p *= 0.62) or in either group independently (Standard r = 0.13, *p *= 0.44; Motivation-enhanced r = -0.01 p = 0.94). Four-week controlled motivation was not associated with weight change in the overall sample (*r *= -0.13; *p *= 0. 31), however in the Standard group four-week controlled motivation was negatively correlated with weight change (*r *= -0.39; *p *= 0. 02). Four week autonomous motivation was associated with weight change in the overall sample (*r *= 0.34; *p *< 0.01). Further analysis revealed this association held true for only the Motivation-enhanced group (*r *= 0.53; *p *< 0.01).

Next, hierarchical regression analysis was conducted to test for the moderating effect of baseline controlled motivation [34]. Baseline controlled motivation was standardized to reduce the possibility of multicollinearity. With the Standard group coded as "-1" and the Motivation-enhanced group coded as "1", in step 1 of the regression, study group and baseline level of controlled motivation were regressed on weight loss. In step 2, the interaction term, group*motivation level, was added (Additional File 2). The interaction term was significant and its addition increased the explained variance (R^2 ^= 0.17), thus confirming the moderating effect of baseline controlled motivation on weight loss.

Two additional regression analyses were also conducted with each study group coded as zero in one of the regression equations in order to determine the independent effect of baseline controlled motivation on each group (Additional File 2). Results from these two regressions show a significant negative slope for the Standard group (B = -0.40, *p *= 0.001) but not for the Motivation-enhanced group (B = 0.01, *p *= 0.99).

**Figure 2:**
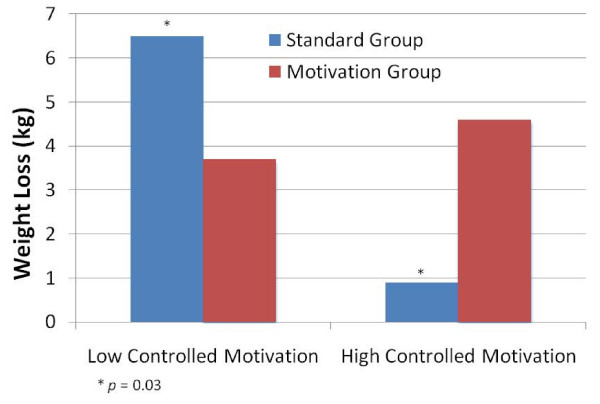
**Weight Loss in Kg by Level of Baseline Controlled Motivation**.

Actual weight loss by level of baseline controlled motivation was also calculated. Participants in the Standard group with low baseline controlled motivation (-1 SD) lost an average of 6.5 kg and participants with high baseline controlled motivation (+ 1 SD) lost an average of 0.93 kg (*p *= 0.03). In the Motivation-enhanced group, participants with low baseline controlled motivation lost an average of 3.7 kg and participants with high baseline controlled motivation lost an average of 4.6 kg (*p *= 0.54) (Figure 2).

## Discussion

Findings from the current study indicate that both groups lost a significant amount of weight and that treatment condition had no effect on the amount of weight loss. The average weight loss was 3.6 ± 3.5 kg and 37% of participants achieved a clinically significant 5% or greater weight loss [37]. Previous short-term behavioral programs with similar populations have produced comparable weight losses. Face-to-face 16-week behavioral weight loss programs have produced weight losses ranging from 2.9 to 5.5 kg [16,38-41], while one 16-week trial of a commercial internet weight loss program produced 0.7 ± 2.7 kg weight loss [41] and another 16-week Internet behavioral weight loss study produced an average weight loss of 4.5 ± 4.6 kg [42].

The motivational treatment did not produce additional weight loss or increase in autonomous motivation in this study. This may have been due to several different factors. The lack of impact of this motivational intervention could have been due to the delivery of the MI in group format. Previously successful interventions have used individual face-to-face MI sessions [17,18]. It is also possible that two sessions may not have been a high enough "dose" to impact weight loss. Previous weight loss interventions that have seen a positive impact of MI on weight loss have included five or more sessions [17,18], however, in our previous work we did find one face-to-face MI session to have a positive impact on autonomous motivation levels and weight loss [42,43]. Finally, while the motivational group sessions were led by a dietitian trained in MI, previous studies incorporated MI sessions led by clinical psychologists or clinical psychology doctoral students [17,18].

A post hoc analysis revealed that treatment condition moderated the effect of baseline controlled motivation on weight loss. In the overall sample, baseline controlled motivation was associated with decreased weight loss. However, those with high baseline controlled motivation lost less than 1 kg in the Standard group compared with 4.6 kg in the Motivation-enhanced intervention. Thus, the MI intervention appeared to buffer the negative effects of controlled motivation on weight loss such that participants in the Motivation-enhanced group had similar weight losses with either high or low baseline controlled motivation. Those with high controlled motivation in the Motivation-enhanced group may have benefitted from the focus of the two motivational sessions on increasing autonomous reasons for weight loss. This exploratory finding is intriguing and suggests a potential benefit to targeting treatment approaches toward motivational profiles when starting a program. As in substance abuse treatment those who feel they are trying to lose weight for external reasons may be more resistant to change, and MI may be helpful in getting these individuals to commit to treatment and make positive changes [12]. Given the potential for weight loss programs to be prescribed by doctors, finding ways to get individuals who may feel externally compelled to start a program to engage in the program and be successful may be useful to explore.

In individuals with low controlled motivation, there appears to be no benefit to adding a MI intervention. In individuals who self-enroll in a weight loss program and who do not feel compelled by external factors to lose weight, the most beneficial strategy may be the one that is most efficient in producing weight loss. Thus, it may be more beneficial to immediately focus on behavioral weight loss skills that are predictive of success rather than spending time trying to increase motivation.

This study did not find that baseline level of autonomous motivation moderated weight loss success for either study group; however, four-week motivation did predict weight loss in the Motivation-enhanced group. Other studies have found autonomous motivation measured after individuals begin behavior change to be more predictive than baseline motivation [6]. This could be because levels of autonomous motivation are high when participants begin a weight loss program but may begin to vary as individuals begin to experience the behavior changes needed for weight loss. When individuals want to make a change, or have more autonomous reasons for change, at the beginning of treatment other approaches including later motivational support or additional social support may be more effective.

## Limitations

The limitations of this study include a small sample size of educated, predominantly white women; therefore, these study results may not be generalizable to other populations. However, this study may suggest a need for future research with larger samples and other populations. Another potential limitation to this study is that it was delivered mainly as an Internet based program. However, the two group dietitian-led sessions were conducted face-to-face and the weekly lessons used for this study were adapted from a face-to-face study [27] and could be used in a face-to-face format in populations which do not have internet access.

## Conclusions

Two sessions of face-to-face motivational intervention as adjunct to an Internet weight loss program did not increase weight loss over two standard weight loss sessions plus the same Internet program over 16-weeks. The motivation-enhanced intervention was significantly more effective than the standard intervention for those who began the study with high levels of controlled motivation.

### Future Directions

More research is needed to determine if more sustained or individualized motivational treatments of this type might increase weight loss in all participants. Future studies could also compare the use of this type of intervention to a standard weight loss intervention among those who may be more likely to have high levels of controlled motivation, such as individuals referred to treatment by a physician. Future studies might also tailor content for those patients with high levels of baseline controlled motivation.

## Competing interests

The authors declare that they have no competing interests.

## Authors' contributions

KW designed and implemented the study and was involved in data analysis and interpretation, and drafting and revision of the manuscript. JG participated in the data analysis and interpretation and drafting and revision of the manuscript. DT participated in the study design, data analysis, and revision of the manuscript. MD participated in the study design, data analysis and interpretation, and revision of the manuscript.

All authors have read and approved the final manuscript.

## Supplementary Material

Additional file 1 **Mean Changes in Outcome Measures from Baseline to 16-week follow-up**. N = 70; Participants in the two groups did not differ on change on any main outcome measures over the 16 weeks. Click here for file

Additional file 2 **Hierarchical Regression Analysis**. This analysis confirms the moderating effect of baseline controlled motivation on weight loss. Click here for file
