# Supplementary material for: The effect of a motivational intervention on weight loss is moderated by level of baseline controlled motivation
Source: Int J Behav Nutr Phys Act. 2010 Jan 22;7:4. doi: 10.1186/1479-5868-7-4 (PMC2821313; doi:10.1186/1479-5868-7-4)
Supplement: Additional file 1 — Mean Changes in Outcome Measures from Baseline to 16-week follow-up. N = 70; Participants in the two groups did not differ on change on any main outcome measures over the 16 weeks. [file 1479-5868-7-4-S1.PDF]

Additional File 1: Mean Changes in Outcome Measures from Baseline to 16-week follow-up for Completers only

	**Standard**<br>**Mean ± SD**<br>(*p*-value for change)	**Motivational**<br>**Mean ± SD**<br>(*p*-value for change)	**Standard vs. Motivational**<br>***p*-value**
**Weight Loss (kg)**	3.4 ± 3.6<br>(p<0.001)	3.9 ± 3.4<br>(p<0.001)	0.57
**Waist Circumference Decrease (cm)**	3.6 ± 5.5<br>(p<0.001)	3.6 ± 5.0<br>(p<0.001)	0.99
**% Body Fat Decrease**	1.8 ± 3.3<br>(p=0.004)	1.6 ± 2.9<br>(p=0.003)	0.85
**Decrease in Energy Intake (kcal/day)**	572 ± 625<br>(p<0.001)	432 ± 470<br>(p<0.001)	0.31
**Decrease in % Fat Intake**	1.6 ± 4.5%<br>(p=0.04)	0.6 ± 5.7%<br>(p=0.56)	0.42
**Increase in Physical Activity (kcal/week)**	124 ± 898<br>(p=0.42)	145 ± 981<br>(p=0.42)	0.93

N= 70; Participants in the two groups did not differ on change on any main outcome measures over the 16 weeks.
