# Supplementary material for: The effect of a motivational intervention on weight loss is moderated by level of baseline controlled motivation
Source: Int J Behav Nutr Phys Act. 2010 Jan 22;7:4. doi: 10.1186/1479-5868-7-4 (PMC2821313; doi:10.1186/1479-5868-7-4)
Supplement: Additional file 2 — Hierarchical Regression Analysis. This analysis confirms the moderating effect of baseline controlled motivation on weight loss. [file 1479-5868-7-4-S2.PDF]

Additional File 2: Hierarchical Regression Analysis

Step and Variable	B	SE B	95% CI	Beta	R^2
Standard Group coded -1 and Motivation Group coded 1					
Step 1					
Group	0.47	0.86	-1.26, 2.19	0.06	
Baseline Controlled Motivation (z score)	-2.00	0.86	-3.72, -0.29	-0.27*	.095*
Step 2					
Group x Baseline Controlled Motivation (z score)	2.01	0.86	0.30, 3.73	0.27*	0.17*
Standard Group coded 0 and Motivation Group coded 1					
Step 1					
Group	0.93	1.73	-2.51, 4.38	0.06	
Baseline Controlled Motivation (z score)	-4.02	1.17	-6.35, -1.69	-0.54*	0.095*
Step 2					
Group x Baseline Controlled Motivation (z score)	4.03	1.71	0.60, 7.45	0.37*	0.17*
Motivation Group coded 0 and Standard Group coded 1					
Step 1					
Group	-0.93	1.73	-4.38, 2.51	-0.06	
Baseline Controlled Motivation (z score)	0.01	1.26	-2.50, 2.52	-0.001	0.095
Step 2					
Group x Baseline Controlled Motivation (z score)	-4.03	1.71	-7.45, -0.60	-0.40*	0.17*

CI= Confidence Interval; *$p<0.05$

This analysis confirms the moderating effect of baseline controlled motivation on weight loss.
